# Diagnostic test performance of screening questions for neurosensory hand-arm vibration injury

**DOI:** 10.1093/occmed/kqaf042

**Published:** 2025-06-17

**Authors:** Albin Stjernbrandt, Ingrid Liljelind, Tohr Nilsson, Hans Pettersson

**Affiliations:** Department of Epidemiology and Global Health, Umeå University, 901 87 Umeå, Sweden; Department of Epidemiology and Global Health, Umeå University, 901 87 Umeå, Sweden; Department of Epidemiology and Global Health, Umeå University, 901 87 Umeå, Sweden; Department of Epidemiology and Global Health, Umeå University, 901 87 Umeå, Sweden

## Abstract

**Background:**

There is a need for efficient screening for hand-arm vibration injury.

**Aims:**

To evaluate the diagnostic test performance of screening questions for neurosensory injury in relation to clinical testing among hand-arm vibration (HAV)-exposed workers.

**Methods:**

HAV-exposed subjects responded to a screening survey on subjective perception of touch, warmth, cold, and vibration, as well as grip strength and manual dexterity. Perception of monofilament, two-point discrimination, temperature rollers, and tuning fork was tested on the index and little fingers of both hands, while grip strength was tested using a hydraulic dynamometer. Diagnostic test performance characteristics were calculated based on crosstabulation of survey responses and outcomes of clinical testing.

**Results:**

Our study recruited 225 subjects with exposure durations between one and 43 years. The sensitivity and specificity for the question about impaired ability to feel touch compared with monofilament was 65% and 71%; impaired ability to feel heat compared with temperature rollers 50% and 75%; impaired ability to feel cold compared with temperature rollers 39% and 77%; impaired ability to feel vibration compared with tuning fork 49% and 84%; reduced grip strength compared with hydraulic dynamometer 83% and 58%; and difficulty fastening buttons compared with two-point discrimination 40% and 76%, respectively.

**Conclusions:**

The diagnostic test performance of the currently used screening survey for neurosensory injury among HAV-exposed workers needs to be improved. Future development should focus on establishing more specific questions, balanced response alternatives, and a more sophisticated interpretation of the responses, possibly using an index made up of several screening questions.

## Teaser

This paper evaluates the diagnostic test performance of a screening survey for neurosensory injury among 225 hand-arm vibration-exposed workers. The survey is currently recommended by the Swedish trade association for occupational health services. Our study shows that the responses to the questions in the survey have limited predictive potential in relation to the outcomes of clinical testing of different sensory modalities. We suggest revising the survey to improve its diagnostic accuracy.

## INTRODUCTION

In Sweden, about 400 000 people work at least one-quarter of the time with vibrating handheld tools [1]. Adverse health effects arising from exposure to hand-arm vibration (HAV) are the most commonly compensated occupational injuries in Sweden [2]. Vascular injuries often manifest as vibration-induced white fingers, a form of secondary Raynaud’s phenomenon [3]. Using a colour chart for self-reporting Raynaud’s phenomenon has been reported to have a sensitivity of 90% and specificity of 100%, using investigator-observed finger blanching as the reference method [4]. When instead using the colour chart as the reference method, the sensitivity and specificity of medical history alone were 88% and 94%, respectively [5]. Thus, vascular HAV injuries are suitable for screening using a survey. Moreover, the ubiquity of mobile phones means that photography can also be used to document vasospastic episodes [6]. Neurosensory injuries from HAV are just as common and include both a diffuse peripheral neuropathy affecting the digital nerves or cutaneous mechanoreceptors and carpal tunnel syndrome [3]. These conditions result in rather similar symptoms, often categorised as positive symptoms/gain-of-function (e.g. paraesthesia or pain) or negative symptoms/loss-of-function (e.g. impaired ability to feel touch or temperature). To assess neurosensory injury, clinical testing of different sensory modalities is often used, sometimes also referred to as bedside or point-of-care testing [7]. Neurosensory injuries are generally not reversible [3] and there is no effective treatment, which emphasizes the need for preventive measures. In this context, early identification of incipient injury is crucial. However, research on the diagnostic value of screening surveys for neurosensory injuries is scarce. In a British study on HAV-exposed workers (*n* = 365), a combination of four questions about tingling and numbness was reported to have a sensitivity of 94% and specificity of 52% [8]. In a later Swedish study on HAV-exposed workers (*n* = 66) that responded to a screening survey about HAV injury, 19 (29%) reported neurosensory symptoms, but only six of these symptomatic subjects were found to have impaired sensory perception during clinical testing [9]. These results suggest that screening questions might be more effective for the detection of vascular than neurosensory HAV injury.

There is Swedish workplace legislation based on the European directive 2002/44/EC to limit the hazardous effects of HAV exposure. If the daily exposure action value for HAV of 2.5 m/s² A(8) is exceeded, any worker shows signs of HAV injury, or there are specific hazards related to the work tasks, periodical medical surveillance should be arranged by the employer and performed by physicians working in the occupational healthcare services (OHS) [10]. These medical check-ups are to be initiated before starting exposure and then repeated every third year. After the sixth year, every other check-up can be substituted with a screening survey, checking for vascular or neurosensory symptoms. When workers report symptoms in the survey, they are to be offered a full medical check-up with clinical testing corresponding to the sensory modalities in which abnormal sensation was reported. The screening questions were originally developed in the late 1980s [11] but have since then been repeatedly modified and are now distributed by the Swedish trade association for OHS, and most of the larger providers use them. However, the diagnostic test performance of these screening questions has not been systematically evaluated.

The main aim of our study was to evaluate the diagnostic test performance of screening questions for neurosensory injury in relation to clinical testing among hand-arm vibration-exposed workers. The secondary aim was to assess the occurrence of self-reported neurosensory symptoms and abnormal clinical findings among hand-arm vibration-exposed workers.

## METHODS

We performed a clinical (non-experimental) cross-sectional study with a convenience sampling method, inviting study participants both directly from companies as well as patients referred to our occupational medicine clinic for suspected HAV injury. Patients were invited regardless of type of suspected injury, i.e. neurosensory, vascular, or musculoskeletal. Symptom assessment regarding sensory perception was determined using forced-choice written questionnaire items and compared to the corresponding neurosensory clinical tests.

The data collection was performed between September 2021 and February 2024 in northern Sweden, in the counties of Västerbotten and Västernorrland. We invited workers, regardless of the presence of self-reported neurosensory symptoms, from six different companies in the construction, tire changing, and manufacturing sectors. In addition, we also recruited patients from the Occupational and Environmental Medicine Clinic at the University Hospital of Umeå, which is the tertiary referral centre for the entire region of northern Sweden, which in turn constitutes about half of the surface area of the country.

The rationale and method of our study have previously been thoroughly described [12]. All participants were asked to complete a screening survey about neurosensory symptoms in the hands. The survey consisted of Likert-type questions beginning with ‘Do you have any of the following?’, and the following statements: ‘Impaired ability to feel touch in fingers/hand?’; ‘Impaired ability to feel heat in fingers/hand?’; ‘Impaired ability to feel cold in fingers/hand?’; ‘Impaired ability to feel vibration in fingers/hand?’; ‘Reduced strength in fingers/hand?’; and ‘Difficulty with fastening buttons?’. The response alternatives were ‘No’, ‘Insignificant’, ‘Somewhat’ and ‘Quite a lot’. As is the current routine among the OHS in Sweden, answering ‘Somewhat’ or ‘Quite a lot’ was considered a positive response.

Clinical neurosensory testing was then performed on the distal volar aspects of the index and little fingers of both hands, using a five-piece Semmes Weinstein monofilament set (Touch-Test®), a two-point discriminator (Touch-Test®), 25 and 40°C temperature rollers (Somedic SenseLab Rolltemp®), a Rydel Seiffer quantitative tuning fork, and a hydraulic grip strength dynamometer (Jamar®). For monofilament, the inability to discriminate 0.4 g target force in at least one out of three challenges was considered abnormal. For two-point discrimination, requiring more than 5 mm separation to be able to discriminate two points in at least two out of three attempts was found to be abnormal. Not being able to distinguish between the warm and cold temperature rollers at the distal phalanx in at least one out of three attempts was considered a sign of impaired temperature discrimination. A mean disappearance threshold below six based on three attempts using the Rydel Seiffer tuning fork was considered abnormal [13]. Maximum grip strength of less than 32 kg for men and 20 kg for women in at least one out of three attempts was also considered abnormal. In analyses of the diagnostic test performance of the survey (including sensitivity, specificity, predictive values, likelihood ratios, and diagnostic odds ratio), having an abnormal clinical finding in at least one out of four fingers was used as the reference method for neurosensory injury. The screening survey item on a specific sensory modality was compared against the clinical test that would reflect the same modality, i.e. impaired ability to feel vibration compared with clinical testing using the tuning fork. Since the positive and negative predictive values are heavily dependent on the prevalence of the condition, these values were calculated based only on subjects recruited directly from companies and not the occupational medicine clinic. Ninety-five percent confidence intervals (95% CI) were calculated assuming a binomial distribution of data. All the results of the screening survey and clinical testing were individually assessed by an occupational physician (AS), and those who were invited directly from companies, reported symptoms, and had abnormal clinical findings were offered a follow-up visit to the occupational medicine clinic to be thoroughly evaluated by the physician. The study protocol was approved by the Swedish Ethical Review Authority (DNR 2021-01463).

## RESULTS

Our study recruited 225 subjects, of which 150 (67%) came directly from companies and 75 from the occupational medicine clinic. The duration of occupational HAV exposure ranged from 1–43 years. Seventeen (8%) were women and 208 men, the mean (SD) age was 40.6 (13.7) years, and the mean (SD) BMI was 27.3 (4.5). Current smoking was reported by 10 (5%) and former smoking by 62 (28%), while current and previous use of oral moist tobacco (snuff) was reported by 96 (43%) and 26 (12%), respectively. Reporting hand symptoms was overall very common (Table 1), and the highest occurrence was found for reduced grip strength, where 99 subjects (44%) reported this symptom somewhat or quite a lot. Experiencing impaired ability to feel vibration was the least common symptom, reported by 57 subjects (26%). Details on the clinical neurosensory testing are presented in Table 2. In brief, impaired perception of touch in at least one out of the four tested fingers was found in 63 subjects (29%), two-point discrimination in 15 (7%), temperature discrimination in 108 (51%), tuning fork in 37 (19%), and reduced grip strength in 12 (5%).

**Table 1.** Occurrence of neurosensory symptoms in the screening survey

Screening question	Response alternatives	*n* (%)
Impaired ability to feel touch in fingers/hand?	No	96 (43)
	Insignificant	37 (16)
	Somewhat	79 (35)
	Quite a lot	13 (6)

Impaired ability to feel heat in fingers/hand?	No	97 (43)
	Insignificant	40 (18)
	Somewhat	66 (29)
	Quite a lot	22 (10)

Impaired ability to feel cold in fingers/hand?	No	112 (50)
	Insignificant	40 (18)
	Somewhat	57 (25)
	Quite a lot	16 (7)

Impaired ability to feel vibration in fingers/hand?	No	122 (55)
	Insignificant	42 (19)
	Somewhat	48 (22)
	Quite a lot	9 (4)

Reduced strength in fingers/hand?	No	87 (39)
	Insignificant	39 (17)
	Somewhat	67 (30)
	Quite a lot	32 (14)

Difficulty with fastening buttons?	No	127 (56)
	Insignificant	38 (17)
	Somewhat	47 (21)
	Quite a lot	13 (6)

**Table 2.** Results of clinical testing for neurosensory injury

Clinical test	Categories	Right hand		Left hand	
		Index finger	Little finger	Index finger	Little finger
***n* (%)**	***n* (%)**	***n* (%)**	***n* (%)**
Monofilament [evaluator size (target force in grams)]	2.83 (0.07)	103 (47)	108 (49)	103 (47)	117 (53)
	3.61 (0.4)	70 (32)	87 (40)	82 (37)	81 (37)
	4.31 (2.0)	46 (21)	24 (11)	32 (15)	20 (9)
	4.56 (4.0)	1 (1)	1 (1)	1 (1)	3 (1)
	6.65 (300)	1 (1)	-	2 (1)	-

Two-point discrimination (mm)	2	99 (46)	46 (22)	106 (50)	41 (19)
	3	72 (34)	88 (41)	61 (29)	90 (42)
	4	21 (10)	45 (21)	31 (15)	48 (22)
	5	16 (7)	24 (11)	9 (4)	26 (12)
	6	6 (3)	9 (4)	6 (3)	9 (4)
	7	1 (1)	1 (1)	-	-
	8	-	1 (1)	-	1 (1)

Temperature discrimination (40/25°C temperature rollers)	Distal phalanx	175 (80)	140 (66)	178 (82)	155 (71)
	Middle phalanx	31 (14)	54 (25)	31 (14)	43 (20)
	Proximal phalanx	11 (5)	16 (8)	9 (4)	16 (7)
	Palm	2 (1)	3 (1)	-	4 (2)
	Wrist	-	-	-	2 (1)

Vibration (Rydel Seiffer tuning fork)	>7	121 (62)	71 (36)	120 (61)	89 (46)
	>6–7	60 (31)	82 (42)	52 (26)	74 (37)
	>5–6	9 (5)	29 (15)	17 (9)	22 (11)
	>4–5	5 (3)	12 (6)	8 (4)	10 (5)
	<4	2 (1)	3 (2)	1 (1)	2 (1)

Maximum grip strength (kg; hydraulic dynamometer) ^a^	<20	4 (2)		4 (2)	
	20–30	12 (5)		15 (7)	
	>30–40	32 (14)		37 (17)	
	>40–50	70 (31)		80 (36)	
	>50–60	68 (31)		56 (25)	
	>60	37 (17)		32 (14)	

^a^According to the currently used cut-offs for men (< 32 kg) and women (< 20 kg), nine men and three women had reduced grip strength in at least one hand.

Using the two highest response alternatives in each screening question as a positive response, the sensitivity and specificity of the item about impaired ability to feel touch was 65% and 71%; heat 50% and 75%; cold 39% and 77%; vibration 49% and 84%; grip strength 83% and 58%; and difficulty with fastening buttons 40% and 76%, respectively. Positive and negative predictive values, positive and negative likelihood ratios, and the diagnostic odds ratio for each item are presented in Table 3. When only using the highest response alternative for each screening question as a positive response, the sensitivity was generally reduced, the specificity increased, and the likelihood ratios for some questions were more decisive (Supplementary Table S1, available as Supplementary data at *Occupational Medicine* online).

**Table 3.** Diagnostic test performance of the screening survey for neurosensory symptoms, using the two highest response alternatives as a positive response

Survey item	Positive response	Clinical test	Abnormal finding	Sensitivity (%)	Specificity (%)	Positive predictive value (%) ^a^	Negative predictive value (%) ^a^	Positive likelihood ratio	Negative likelihood ratio	Diagnostic odds ratio
	*n* (%)		*n* (%)	Estimate (95% CI)	Estimate (95% CI)	Estimate (95% CI)	Estimate (95% CI)	Estimate (95% CI)	Estimate (95% CI)	Estimate
Impaired ability to feel touch	92 (41)	Monofilament any finger	63 (29)	65 (53–76)	71 (64–78)	33 (21–48)	91 (84–95)	2.3 (1.7–3.1)	0.5 (0.4–0.7)	4.6
										
Impaired ability to feel heat	88 (39)	Temperature rollers any finger	108 (51)	50 (41–59)	75 (66–82)	66 (51–78)	60 (50–68)	2.0 (1.4–2.9)	0.7 (0.5–0.8)	3.0
										
Impaired ability to feel cold	73 (32)	Temperature rollers any finger	108 (51)	39 (30–48)	77 (68–84)	58 (41–74)	56 (47–64)	1.7 (1.1–2.6)	0.8 (0.7–1.0)	2.1
										
Impaired ability to feel vibration	57 (26)	Tuning fork any finger	37 (19)	49 (33–64)	84 (78–89)	23 (10–43)	91 (84–95)	3.1 (1.9–5.1)	0.6 (0.4–0.8)	5.1
										
Reduced grip strength	99 (44)	Hydraulic dynamometer any hand	12 (5)	83 (55–95)	58 (52–65)	6 (2–16)	98 (96–100)	2.0 (1.5–2.7)	0.3 (0.1–1.0)	6.9
										
Difficulty with fastening buttons	60 (27)	Two-point discrimination any finger	15 (7)	40 (20–64)	76 (70–82)	4 (1–21)	99 (97–100)	1.7 (0.9–3.3)	0.8 (0.5–1.2)	2.1
										
Any of the above	146 (66)	Any of the above	107 (60)	68 (59–76)	49 (38–61)	60 (49–70)	51 (39–62)	1.4 (1.0–1.8)	0.7 (0.5–0.9)	2.1

^a^Calculated for subjects invited directly from companies only (N = 150).

## DISCUSSION

Among workers exposed to HAV, both self-reported neurosensory symptoms as well as abnormal clinical findings were common. The sensitivity (95% CI) of different screening questions to predict abnormal clinical test results ranged from 39 (30–48) to 83 (55–95)%, while the specificity ranged from 58 (52–65) to 84 (78–89) %. Between 26 and 44% reported symptoms in the survey, and abnormal clinical findings were found in 5 to 51%, depending on modality.

When compared to the diagnostic test performance of a screening survey for vibration-induced white fingers, with a sensitivity and specificity of about 90% [4], the precision of the screening questions for neurosensory injury, as evaluated in our study, was poorer. Using only the highest response alternative as a positive response increased the specificity of the screening questions, at the expense of sensitivity (Supplementary Table S1, available as Supplementary data at *Occupational Medicine* online). It could be argued that a screening survey should be designed to have a high sensitivity and tolerate a proportion of false-positive responses that proceed to a full medical check-up by an occupational physician before being correctly classified. Therefore, we do not support a change in the interpretation of the answers to only consider the highest response alternative as a positive response. Rather, the questions themselves may need revision to better reflect the presence of neurosensory HAV injury. When interviewing the study participants, it was evident that the questions were commonly misinterpreted. To begin with, the temporality was not well understood. Some subjects had reported neurosensory symptoms, but when confronted with the response, clarified that it related to transient and not current symptoms. Moreover, when reflecting on the current status, some subjects related their hand function to their previous status (e.g. having lower grip strength now compared to previously), while others interpreted the question as a comparison against a wider context (e.g. being stronger or weaker than coworkers or people in general). Another issue was that the anatomical distribution of symptoms was not weighted, e.g. experiencing impaired perception in a small part of a finger as opposed to having the same sensation in the whole hand. The response alternatives could also be improved, as they were found to be skewed towards negative alternatives. As pointed out by several study participants, ‘No’, ‘Insignificant’, and ‘Somewhat’ all reflected absence or rather low intensity of symptoms. In contrast, ‘Quite a lot’ was felt to indicate a high intensity of symptoms.

In our study, the positive likelihood ratios for survey items to predict clinical test outcomes ranged from 1.4 to 3.1, and the negative likelihood ratios from 0.3 to 0.8. The further the likelihood ratios are from 1, the stronger the evidence for the presence or absence of injury. In medicine, a positive likelihood ratio exceeding 10 can be considered to provide strong evidence to rule in injury, while the corresponding figure for strongly ruling out injury would be a negative likelihood ratio below 0.1 [14]. Altogether, we interpret that the survey questions did not provide sufficient support for the presence or absence of abnormal clinical findings. The diagnostic odds ratios in our study ranged from 2.1 to 6.9. This measure indicates test performance that is not generally dependent on the prevalence of the condition. The diagnostic odds ratio ranges from zero to infinity, and a value of 1 indicates that the test does not discriminate between those with and without a disorder, while a higher value suggests a better discriminatory test performance [15]. Here, our interpretation of the diagnostic odds ratio is that most screening questions did not discriminate properly between those with normal or abnormal clinical findings. Finally, other attributes of screening tests are also important to consider, but not the focus of our study, such as validity and test-retest reliability [16].

Comparison with previous studies on the occurrence of neurosensory symptoms among HAV-exposed workers is sometimes hampered by the fact that different questionnaire items have been used [8] or responses not reported separately [9]. However, we have previously tested the same screening questions and response alternatives as used in our current study on a sample of the general population (*N* = 12 627) that were of working age (18–70 years) [17]. In this sample, impaired perception of touch was reported by 7%, heat by 4%, cold by 5%, and reduced grip strength by 17%. However, only about 18% of the subjects in the cohort were occupationally exposed to HAV [18]. In a clinical study by Tekavec et al. on Swedish HAV-exposed carpenters (*n* = 193), the same screening questions were used, and impaired perception of touch was reported by 15%, heat by 12%, cold by 10%, and reduced grip strength by 18% [19]. Thus, it appears that the subjects in our study reported a much higher occurrence of neurosensory symptoms, about 26–44%, depending on modality. This could be explained by the fact that we recruited part of the sample from the occupational medicine clinic, where symptomatic subjects are likely more prevalent since they were referred due to suspected HAV injury. When the subjects in the study by Tekavec et al. were examined clinically, an inability to discriminate monofilament (target force 0.4 grams) was found in 31%, two-point discrimination (5 mm) in 9%, and temperature rollers (applied at the middle phalanges) in 4% (heat) to 6% (cold). In comparison, roughly 29% of subjects in our study were unable to distinguish the same kind of monofilament (target force 0.4 grams), 7% two-point discrimination (5 mm), and 16% temperature rollers (at the middle phalanges) in at least one finger. The general impression was therefore that the subjects in our study were more symptomatic according to the responses to the screening questions, but did not show a much higher occurrence of impaired sensory function in the hands when tested. The screening survey has also been tested by us on a cohort of Swedish and Norwegian open-pit miners (*n* = 260), where 29% were currently HAV-exposed [20]. In this study, impaired perception of touch was reported by 8%, heat by 6%, cold by 2%, and reduced grip strength by 16%. When clinically examined, an inability to distinguish monofilament (target force 0.4 grams) was found in 2%, temperature rollers (at the distal phalanges) in 34%, and tuning fork (128 Hz) in 7%. In comparison, neurosensory symptoms were again more commonly reported in our present study. Apart from rather similar figures for temperature discrimination, the subjects in our present study also had a higher proportion of abnormal clinical findings.

There are several potential methodological issues to address. First, the diagnostic test performance of the screening questions is inherently linked to the occurrence of neurosensory injury within the studied population, i.e. the pre-test probability. Therefore, we encourage further studies on other samples of HAV-exposed workers to get a broader perspective on the test performance in other settings. When planning our study, we anticipated that only inviting study participants directly from companies might have resulted in a sample of mostly asymptomatic subjects without abnormal clinical findings, which would have made it hard to evaluate the usefulness of the screening survey. Therefore, we opted for a mixed sample with a minor proportion of subjects from the occupational medicine clinic, who we knew would be symptomatic to some extent. We also believe that this mixed sample could be considered to be representative of the population undergoing screening for HAV injury in the OHS. We used a convenience sampling technique to reach the intended sample size. With this approach, there was a risk of selection bias, where company-recruited subjects may have been more prone to participate in our study if symptomatic. We only investigated negative symptoms (loss-of-function), although positive symptoms can also be important indicators of emerging neurosensory affliction. For study participants invited directly from companies, all were examined by the same two researchers (IL and HP), which likely reduced the interobserver-variability. For the subset of subjects recruited from the occupational medicine clinic, there were several different occupational physicians involved, and there is a risk that there could have been greater dissimilarities in the examination procedure. However, we had written protocols for all examination techniques, which served to reduce this risk. The same examination equipment was used in both settings and calibrated according to the instructions by the manufacturers. Therefore, we do not believe that any major systematic bias could have been introduced in the clinical testing. Finally, since nerve conduction studies were not performed, we could not determine the potential role of nerve entrapment syndromes in relation to neurosensory symptoms and clinical tests.

To conclude, the diagnostic test performance of the currently used screening survey for neurosensory injury among HAV-exposed workers needs to be improved. Future development should focus on establishing more specific questions (e.g. nocturnal dysesthesia), balanced response alternatives, and a more sophisticated interpretation of the responses, possibly using an index made up of several screening questions.

**Key learning points**

**What is already known**

- Hand-arm vibration injury is the most commonly compensated occupational injury in Sweden.
- After six years, every other periodical medical surveillance is commonly substituted with a screening survey instead of an appointment with an occupational physician.
- Though in current use nationally, the diagnostic test performance of the screening questions has not previously been evaluated.

**What this study adds**

- Our study demonstrates that self-reported neurosensory symptoms are very common but not necessarily associated with abnormal results during clinical testing.
- There is ambiguity in both the questionnaire items and the response alternatives in the screening survey.
- At present, the screening survey cannot be considered to adequately substitute an appointment with an occupational physician.

**What impact can this study have on practice or policy**

- We advocate an extensive revision of the screening survey for hand-arm vibration-exposed workers.
- Occupational physicians that are confronted with positive responses in this screening survey should interpret them cautiously.

## Supplementary Material

kqaf042_suppl_Supplementary_Tables_S1

## Data Availability

The dataset used in our study can be made available upon reasonable request to the corresponding author.
