# Supplementary material for: Diagnostic test performance of screening questions for neurosensory hand-arm vibration injury
Source: Occup Med (Lond). 2025 Jun 17;75(3-4):196–202. doi: 10.1093/occmed/kqaf042 (PMC12257927; doi:10.1093/occmed/kqaf042)
Supplement: kqaf042_suppl_Supplementary_Tables_S1 [file kqaf042_suppl_supplementary_tables_s1.docx]

**Article title:** Diagnostic test performance of screening questions for neurosensory hand-arm vibration injury

**Journal name:** Occupational Medicine

**Authors:** Albin Stjernbrandt ^1^, Ingrid Liljelind ^1^, Tohr Nilsson ^1^, Hans Pettersson ^1^

**Affiliation:** ^1^ Department of Epidemiology and Global Health, Umeå University, Umeå, Sweden

**E-mail address of the corresponding author:** [albin.stjernbrandt@umu.se](mailto:albin.stjernbrandt@umu.se)

**Supplementary Table S1** Diagnostic test performance of the screening survey for neurosensory symptoms, using only the highest response alternative (“Quite a lot”) as a positive response

| **Survey item** | **Positive response** | **Clinical test** | **Abnormal finding** | **Sensitivity (%)** | **Specificity (%)** | **Positive predictive value (%) ^a^** | **Negative predictive value (%) ^a^** | **Positive likelihood ratio** | **Negative likelihood ratio** | **Diagnostic odds ratio** |
| --- | --- | --- | --- | --- | --- | --- | --- | --- | --- | --- |
|  | **n (%)** |  | **n (%)** | **Estimate (95% CI)** | **Estimate (95% CI)** | **Estimate (95% CI)** | **Estimate (95% CI)** | **Estimate (95% CI)** | **Estimate (95% CI)** | **Estimate** |
| Impaired ability to feel touch | 13 (6) | Monofilament any finger | 63 (29) | 14 (8–25) | 98 (95–99) | 50 (15–85) | 85 (78–90) | 7.5 (2.1–26.5) | 0.9 (0.8–1.00) | 8.7 |
| Impaired ability to feel heat | 22 (10) | Temperature rollers any finger | 108 (51) | 12 (7–20) | 94 (88–97) | 60 (31–83) | 54 (45–62) | 2.1 (0.8–5.3) | 0.9 (0.9–1.0) | 2.2 |
| Impaired ability to feel cold | 16 (7) | Temperature rollers any finger | 108 (51) | 11 (7–18) | 98 (93–100) | 83 (44–97) | 54 (46–62) | 5.8 (1.3–25.2) | 0.9 (0.8–1.0) | 6.4 |
| Impaired ability to feel vibration | 9 (4) | Tuning fork any finger | 37 (19) | 5 (2–18) | 99 (96–100) | 33 (6–79) | 89 (83–93) | 4.2 (0.6–29.5) | 1.0 (0.9–1.0) | 4.3 |
| Reduced grip strength | 32 (14) | Hydraulic dynamometer any hand | 12 (5) | 33 (14–61) | 87 (81–91) | - ^b^ | 98 (94–99) | 2.5 (1.0–6.0) | 0.8 (0.5–1.2) | 3.2 |
| Difficulty with fastening buttons | 5 (3) | Two-point discrimination any finger | 15 (7) | 20 (7–45) | 96 (92–98) | - ^b^ | - ^b^ | 4.9 (1.5–16.7) | 0.8 (0.7–1.1) | 5.9 |
| Any of above | 42 (19) | Any of above | 107 (60) | 22 (15–30) | 92 (83–96) | 80 (58–92) | 49 (41–58) | 2.5 (1.1–5.9) | 0.9 (0.8–1.0) | 2.9 |

^a^ Calculated for subjects invited directly from companies only (N=150)
^b^ Not possible to calculate because of empty cells
